# Efficacy and Safety of Filgotinib for the Treatment of Perianal Fistulising Crohn’s Disease [DIVERGENCE 2]: A Phase 2, Randomised, Placebo-controlled Trial

**DOI:** 10.1093/ecco-jcc/jjae003

**Published:** 2024-02-16

**Authors:** Walter Reinisch, Jean-Frederic Colombel, Geert R D’Haens, Jordi Rimola, Tomasz Masior, Matt McKevitt, Xuehan Ren, Adrian Serone, David A Schwartz, Krisztina B Gecse

**Affiliations:** Department of Internal Medicine III, Medical University of Vienna, Vienna, Austria; Dr Henry D. Janowitz Division of Gastroenterology, Icahn School of Medicine at Mount Sinai, New York, NY, USA; Department of Gastroenterology and Hepatology, Amsterdam University Medical Centers, Amsterdam, The Netherlands; Inflammatory Bowel Disease Unit, Department of Radiology, Hospital Clinic of Barcelona, Barcelona, Spain; Galapagos NV, Mechelen, Belgium; Gilead Sciences, Foster City, CA, USA; Gilead Sciences, Foster City, CA, USA; Gilead Sciences, Foster City, CA, USA; Division of Gastroenterology, Hepatology and Nutrition, Vanderbilt University Medical Center, Nashville, TN, USA; Department of Gastroenterology and Hepatology, Amsterdam University Medical Centers, Amsterdam, The Netherlands

**Keywords:** Filgotinib, perianal fistulising Crohn’s disease, Janus kinase inhibitor

## Abstract

**Background and Aims:**

There is an unmet need in the treatment of perianal fistulising Crohn’s disease [PFCD]. This study evaluated the efficacy and safety of the Janus kinase 1 preferential inhibitor, filgotinib, for the treatment of PFCD.

**Methods:**

This phase 2, double-blind, multicentre trial enrolled adults with PFCD and prior treatment failure. Participants were randomised [2:2:1] to receive filgotinib 200 mg, filgotinib 100 mg, or placebo, once daily orally for up to 24 weeks. The primary endpoint was combined fistula response (reduction from baseline of at least one draining external opening determined by physical assessment, and no fluid collections >1 cm on pelvic magnetic resonance imaging [MRI]) at Week 24.

**Results:**

Between April 2017 and July 2020, 106 individuals were screened and 57 were randomised. Discontinuations were lowest in the filgotinib 200 mg group (3/17 [17.6%] versus 13/25 [52.0%] for filgotinib 100 mg and 9/15 [60.0%] for placebo). The proportion of participants who achieved a combined fistula response at Week 24 was 47.1% (8/17; 90% confidence interval [CI] 26.0, 68.9%) in the filgotinib 200 mg group, 29.2% [7/24; 90% CI 14.6, 47.9%] in the filgotinib 100 mg group, and 25.0% [3/12; 90% CI 7.2, 52.7%] in the placebo group. Serious adverse events occurred more frequently with filgotinib 200 mg (5/17 [29.4%]) than with placebo (1/15 [6.7%]). There were no treatment-related serious adverse events or deaths.

**Conclusions:**

Filgotinib 200 mg was associated with numerical reductions in the number of draining perianal fistulas based on combined clinical and MRI findings compared with placebo, and was generally well tolerated [NCT03077412].

## 1. Introduction

Crohn’s disease [CD] is a relapsing and remitting form of inflammatory bowel disease characterised by chronic, transmural inflammation that can progress to complications such as stricturing and/or penetrating disease.^1^ Perianal fistulas affect 11–38% of patients with CD.^2^ They are a major clinical challenge in the management of CD, owing to severe impairments in patients’ quality of life, limited treatment options, and a frequent need for surgery.^2^

Perianal fistulas can be categorised based on their anatomy as simple or complex. A simple fistula is low [superficial, inter-sphincteric, or trans-sphincteric] with a single external opening [EO] and no rectovaginal involvement, abscesses, or anorectal stricture.^3^ All other fistulas are classified as complex fistulas and are more difficult to treat, with decreased healing rates and less successful outcomes than simple fistulas.^3^

Perianal fistulising CD [PFCD] is a debilitating disease, and long-term efficacy has rarely been documented for drug or surgical therapies. In the clinic, a ‘top-down’ approach is often taken, beginning with tumour necrosis factor [TNF] inhibitor therapy in combination with seton draining, antibiotics, and often thiopurines.^4^ Of the targeted therapies approved for the treatment of active CD, only infliximab has been shown in randomised, placebo-controlled clinical trials to significantly reduce the number of draining fistulas, as assessed via physical examination, in patients with abdominal or perianal fistulas.^5,6^ Although significant versus placebo, the maintenance of complete closure with infliximab was, however, low.^6^ By Week 54, 19% [19/98] of those in the placebo group had complete closure versus 36% [33/91] in the infliximab group.^6^

Long-term healing of patients with PFCD may be improved when TNF inhibitor therapy is combined with surgical fistula closure.^7^ After 18 months of TNF inhibitor treatment, fistula closure [based on MRI assessment] was significantly more common in patients who were surgically treated (12/38 [32%]) than in those who only received TNF inhibitor treatment (5/56 [9%], *p* = 0.005).^7^ However, although surgical closure may improve MRI healing, only a select group of patients are amenable for surgery.

Darvadstrocel [a human, allogeneic, mesenchymal, stem cell therapy] was also shown in randomised, placebo-controlled trials to be efficacious for treatment-refractory complex perianal fistulas in patients with CD, based on the first ever use of a composite endpoint of clinical and magnetic resonance imaging [MRI] assessments. Darvadstrocel was recently approved for the treatment of perianal fistulas that have shown an inadequate response to at least one conventional or biologic therapy, but only in adults with inactive or mildly active local luminal CD.^8–10^

Given the limited evidence for the efficacy of infliximab according to the current, standard, composite endpoint of clinical and MRI assessments, and the fact that administration of darvadstrocel is not indicated for the treatment of perianal fistulas in patients with more than mildly active local luminal CD, there is a pressing need to explore further potential therapies in patients with PFCD.

Janus kinase [JAK] inhibitors are small molecule drugs that block one or more JAK enzymes [JAK1–3 and tyrosine kinase 2] to reduce cytokine signalling and, thus, inflammation.^11^ It has been hypothesised that targeted inhibition of JAK1 may avoid undesired downstream effects of pan-JAK inhibition, while providing the same or better efficacy.^12,13^ Filgotinib is a second-generation, once-daily, oral, JAK1 preferential inhibitor in development for the treatment of multiple inflammatory diseases, and has recently been approved for the treatment of ulcerative colitis in the European Union, Japan, and the UK.^14,15^ Data from the phase 2 FITZROY study [NCT02048618] suggest that filgotinib could be efficacious in the treatment of both luminal and perianal CD.^16^ The exploratory DIVERGENCE 2 study aimed to evaluate the efficacy and safety of filgotinib in participants with PFCD.

## 2. Materials and Methods

### 2.1. Study design and participants

DIVERGENCE 2 [ClinicalTrials.gov identifier: NCT03077412] was a 24-week, phase 2, randomised, double-blind, placebo-controlled study conducted at 27 centres across nine countries [Austria, Belgium, Canada, France, Germany, Hungary, Italy, UK, and USA] between April 2017 and February 2021. The study protocol and amendments were approved by the institutional review board or independent ethics committee for each centre. All participants provided written informed consent.

Participants were aged 18–75 years and were men or non-pregnant, non-lactating women, who had a diagnosis of CD for at least 3 months. Participants were also required to have: a CD Activity Index [CDAI] score of ≤300 at screening; moderately to severely active PFCD, defined as the presence of one to three perianal EOs with drainage [spontaneous or on compression] at randomisation and for at least 4 weeks before screening; and a previous, inadequate, clinical response or loss of response [both defined as active disease despite treatment], or intolerance to antibiotics, immunomodulators, or TNF inhibitors. Participants with non-cutting, perianal setons had these removed at least 14 days before randomisation.

### 2.2. Randomisation and masking

The principal investigators at each study site enrolled participants. Eligible participants were randomly assigned using an interactive web response system in a 2:2:1 ratio to receive filgotinib 200 mg and placebo-to-match [PTM] filgotinib 100 mg, filgotinib 100 mg and PTM filgotinib 200 mg, or PTM filgotinib 200 mg and PTM filgotinib 100 mg.

Male participants from the USA who were not dual refractory [where dual refractory was defined as previous failure of at least one TNF inhibitor and either previous failure of vedolizumab induction therapy or treatment with vedolizumab at randomisation] were randomised in a 2:1 ratio to receive either filgotinib 100 mg or PTM.

Treatment assignments were stratified according to simple or complex anatomy of draining perianal fistulas at screening [determined by MRI]; presence of moderately to severely active proctitis, defined using the proctitis Simple Endoscopic Score for CD [SES-CD, defined as the sum of ulcer size and ulcerated surface in rectum and anal canal segments] as a score of > 2; and prior exposure to a TNF inhibitor [replaced with stratification according to concomitant vedolizumab therapy at randomisation on April 11, 2019, as per protocol Amendment 4]. Treatment with biologics other than vedolizumab was not allowed during the study or in the 8 weeks before screening.

During the randomised phase, participants and all personnel directly involved in the conduct of the study were blinded to treatment assignment. The study drug was dispensed to the participants by the study pharmacist or a designee in a blinded fashion. PTM filgotinib 200 mg and 100 mg tablets were identical in appearance to the respective tablets containing active treatment.

### 2.3. Procedures and assessments

Filgotinib tablets [100 mg and 200 mg] and PTM filgotinib tablets [100 mg and 200 mg] were administered once daily, orally, with or without food, for 24 weeks. Each participant was given instructions to maintain approximately the same daily time of administration, to ensure a similar dosing interval between study drug doses.

Participants were allowed the following concomitant treatments: oral 5-aminosalicyclic acids, azathioprine, 6-mercaptopurine, methotrexate, or oral steroid therapy [prednisone at a dose of ≤20 mg/day or budesonide at a dose of ≤6 mg/day] provided doses were stable for ≥ 4 weeks before randomisation and for ≥10 weeks after randomisation; antidiarrhoeals for chronic diarrhoea [stable doses were encouraged]; occasional use of nonsteroidal anti-inflammatory drugs for transient symptoms (daily aspirin use [up to 162.5 mg] was allowed for cardiovascular prophylaxis); antibiotics for the treatment of PFCD [eg, metronidazole and ciprofloxacin] provided doses were stable for ≥2 weeks before randomisation and for ≥10 weeks after randomisation; and vedolizumab therapy [300 mg intravenously] following protocol Amendment 4 [April 11, 2019] for the treatment of concurrent luminal disease activity provided the dose was stable for ≥14 weeks before randomisation.

Participants receiving concomitant vedolizumab therapy at randomisation were required to continue vedolizumab treatment during study participation and were not allowed concurrent azathioprine, 6-mercaptopurine, or methotrexate.

For participants receiving concomitant steroids, steroid tapering began post-randomisation at Week 10. Prednisone doses were reduced by 2.5 mg/week up to 5.0 mg/week [or equivalent taper if not prednisone] until the participants were no longer receiving steroids. Participants receiving budesonide had their daily dose reduced by 3.0 mg every 3 weeks. If participants experienced a return of luminal CD symptoms during steroid tapering, their dose was increased or restarted. If a participant required a steroid dose that exceeded their baseline dose, treatment was considered to have failed for all clinical endpoints, but the participant was permitted to remain in the study.

Participants who were CD or PFCD non-responders at Week 10 or who met disease worsening criteria after Week 10 were discontinued from the study. CD non-responders were defined as participants who either had a baseline CDAI score of ≥220 and did not achieve a ≥70-point reduction in the CDAI score at any time up to and including Week 10, or had a baseline CDAI score of <220 and an increase in the CDAI score of ≥100 from baseline, with a CDAI score of ≥220 at Week 10. PFCD non-responders were defined as participants who met the following Perianal CD Activity Index [PDAI] symptom subscore criteria: ‘Discharge’ subscore of >1 and a ≥1-point increase from baseline at Week 6 and Week 10, or ‘Pain/restriction of activities’ subscore of >1 and a ≥1-point increase from baseline at Week 6 and Week 10. Worsening luminal disease was defined as a ≥100-point increase in CDAI score from the Week 10 value, with a CDAI score of ≥220 at two consecutive visits.

The full schedule of study assessments is presented in Supplementary Table 1. Perianal assessment, including completion of the Perianal Fistula Assessment Worksheet, was performed at screening and throughout the study. Centrally read pelvic MRI examinations were conducted at screening to determine the anatomy of draining perianal fistulas and categorise them as simple or complex, and at baseline and Week 24 to assess the size of fluid collections. A centrally read, flexible sigmoidoscopy of the rectum and anal canal was conducted at screening and Week 24, to determine the severity of proctitis using the modified proctitis SES-CD score. The PDAI score, CDAI score, 11-point numerical rating scale [NRS] score for perianal pain, and inflammatory biomarkers were assessed throughout the study as per the schedule [Supplementary Table 1].

Treatment-emergent adverse events [TEAEs] were continually monitored and coded using the Medical Dictionary for Regulatory Activities version 23.1. An independent cardiovascular safety endpoint adjudication committee [Supplementary Table 2] was formed to periodically review and adjudicate all potential major adverse cardiovascular events [MACE] and thromboembolic events, in a blinded manner.

### 2.4. Outcomes

The primary endpoint was the proportion of participants who achieved a combined fistula response, defined as a reduction of at least one from baseline in the number of draining EOs, as determined by physical assessment, and no fluid collections of >1 cm in at least two dimensions on pelvic MRI, at Week 24 [among participants who had at least one draining EO at baseline]. Secondary endpoints included the proportion of participants who achieved combined fistula remission, defined as closure of all draining EOs present at baseline, as determined by physical assessment, and no fluid collections of >1 cm in at least two dimensions on pelvic MRI, at Week 24 [among participants who had at least one draining EO at baseline]; the time to a clinical fistula response, defined as a reduction of at least one from baseline in the number of draining EOs that were present at baseline [among participants who had at least one draining EO at baseline]; the time to clinical fistula remission, defined as closure of all EOs that were draining at baseline [among participants who had at least one draining EO at baseline]; the proportion of participants achieving proctitis remission, defined as a modified proctitis SES-CD score of 0, at Week 24 [only in those who had moderately to severely active proctitis [modified proctitis SES-CD score of >2] at baseline]; and safety endpoints, including TEAEs of interest (infections, malignancies [excluding nonmelanoma skin cancers], nonmelanoma skin cancers, gastrointestinal perforations, positively adjudicated MACE, and thromboembolic events], clinical laboratory analyses, measurements of vital signs, electrocardiograms, and physical examinations. Exploratory endpoints included changes from baseline in PDAI scores, CDAI scores (only in participants who had moderately active luminal disease [CDAI score of ≥ 220] at baseline), 11-point NRS scores for perianal pain, and inflammatory biomarkers (serum C-reactive protein [CRP], faecal calprotectin, and faecal lactoferrin).

The proportion of participants with a ≥50% reduction in modified proctitis SES-CD score at Week 24 [assessed only in the subgroup with moderately to severely active proctitis at baseline] was summarised in a post hoc analysis.

### 2.5. Statistical analysis

No formal sample size calculations were performed, owing to the exploratory nature of the study. Approximately 75 participants were planned to be randomised: 30 to each filgotinib dose group and 15 to the placebo group. This sample size was considered adequate to assess the efficacy and safety of filgotinib in an exploratory and descriptive manner. No formal hypothesis testing was performed.

The full analysis set included all randomised participants who took at least one dose of study drug, and was the primary analysis set for efficacy analyses. The biomarker analysis set included all participants in the full analysis set who had a baseline value and at least one post-baseline value for at least one of the three inflammatory biomarkers assessed in the trial.

The numbers and proportions of participants with a combined fistula response at Week 24 for each treatment group were summarised with corresponding 90% exact confidence intervals [CIs] by the Clopper–Pearson method, which assumes a binomial distribution of the data and is considered a more conservative approach than a chi square test for the small sample size in this study. Binary secondary efficacy endpoints were analysed in the same manner. For time-to-event secondary efficacy endpoints, the Kaplan–Meier method was used to obtain summary statistics for each treatment group. Descriptive statistics were used to summarise the absolute values and change from baseline values in CDAI scores, 11-point NRS scores [for perianal pain], and inflammatory biomarkers. Continuous endpoints were summarised using descriptive statistics, by treatment group, and analysis visit.

Participants with treatment failure, defined as the commencement or dose escalation of potentially effective non-study treatment for PFCD, had all efficacy data censored [ie, set to missing]. Missing values at Week 24 in participants who completed the Week 24 visit were imputed for the derivation of combined fistula response and combined fistula remission rates. For participants who completed the Week 24 visit, if clinical fistula assessment data were missing at Week 24, the number of draining EOs was imputed by the last observation carried forward method using the number of draining EOs observed [among draining EOs at baseline only] at the last available visit. Similarly, for participants who completed the Week 24 visit, if MRI data were missing at Week 24, the absence of a fluid collection of >1 cm was imputed based on whether there was an absence of a fluid collection of >1 cm at baseline [derived from the size of fluid collections at baseline]. If participants did not complete the Week 24 visit [including participants with treatment failure, non-responders at Week 10, and those with worsening luminal disease from Week 10 to Week 24], they were not considered to have met the endpoints of combined fistula response and combined fistula remission [ie, non-responder imputation].

Participants with insufficient measurements to determine proctitis endpoints [including participants with treatment failure, non-responders at Week 10, and those with worsening luminal disease from Weeks 10–24] were not considered to have met those endpoints [ie, non-responder imputation]. In participants with an unknown clinical fistula response [or unknown clinical fistula remission], the time to a clinical fistula response [or clinical fistula remission] was censored at the last perianal fistula assessment date.

Safety analyses were performed using the safety analysis set, which included all participants who took at least one dose of study drug. TEAE data were summarised using descriptive statistics.

Statistical analyses were performed using SAS software version 9.4 [SAS Institute, Cary, NC, USA].

## 3. Results

**Figure 1.**
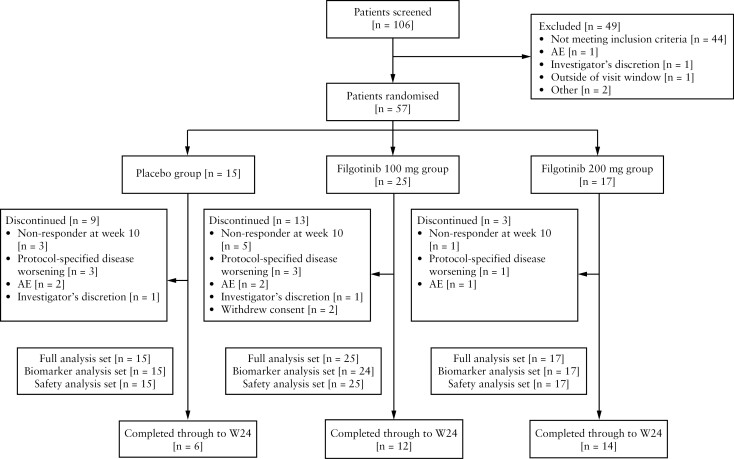
Trial profile. The safety analysis set included all participants who took at least one dose of study drug. The full analysis set [primary analysis set for efficacy analyses] included all randomised participants who received at least one dose of study drug. AE, adverse event; W24, Week 24.

Between April 6, 2017, and July 30, 2020, 106 individuals were screened, of whom 57 were randomised: 17 to the filgotinib 200 mg group, 25 to the filgotinib 100 mg group, and 15 to the placebo group [Figure 1]. Discontinuations were lowest in the filgotinib 200 mg group (3/17 [17.6%] versus 13/25 [52.0%] for filgotinib 100 mg and 9/15 [60.0%] for placebo). The most common reasons for discontinuation were non-response at Week 10 (15.8% [9/57]), protocol-specified disease worsening (12.3% [7/57]), and AEs (8.8% [5/57]). Study enrolment was prematurely terminated owing to low recruitment rates during the COVID-19 pandemic.

Baseline demographics and clinical characteristics were broadly similar across the treatment groups [Table 1]. The mean [SD] CDAI score was 192 [62.3], and 71.9% [41/57] of participants had active luminal disease. Overall, 91.2% [52/57] of participants had complex perianal fistulas and 64.9% [37/57] had prior treatment failure with a TNF inhibitor. Concomitant use of vedolizumab was reported for only 7.0% [4/57] of participants and was balanced across the treatment groups. Concomitant use of corticosteroids, and/or immunomodulators, and antibiotics relevant to fistula healing, was also balanced across the treatment groups.

**Table 1:** Baseline characteristics [safety analysis set].

	Placebo [*n* = 15]	Filgotinib 100 mg [*n* = 25]	Filgotinib 200 mg [*n* = 17]
Age, years	39 [11.8]	41 [14.0]	39 [11.2]
Female	4 [26.7%]	10 [40.0%]	9 [52.9%]
Smoking status			
Former	4 [26.7%]	6 [24.0%]	5 [29.4%]
Current	5 [33.3%]	2 [8.0%]	4 [23.5%]
Never	6 [40.0%]	17 [68.0%]	8 [47.1%]
Duration of PFCD, years	7.5 [7.9]	11.9 [11.1]	10.3 [8.3]
CDAI score	190 [57.9]	194 [67.1]	190 [62.4]
Active luminal disease^a^	11 [73.3%]	17 [68.0%]	13 [76.5%]
Proctitis SES-CD score	2 [1.5]	2 [2.0]	2 [1.7]
Moderately to severely active proctitis	7 [46.7%]	13 [52.0%]	10 [58.8%]
PDAI score	7 [3.1]	8 [3.4]	9 [3.1]
Complex perianal fistulas on MRI^b^	13 [86.7%]	22 [88.0%]	17 [100.0%]
Number of draining perianal fistulas at baseline			
0	3 [20.0%]	1 [4.0%]	0
1	8 [53.3%]	15 [60.0%]	13 [76.5%]
2	2 [13.3%]	7 [28.0%]	4 [23.5%]
3	2 [13.3%]	2 [8.0%]	0
Fluid collections > 1 cm on pelvic MRI	0	3 [12.0%]	5 [29.4%]
History of anal stenosis	1 [6.7%]	1 [4.0%]	4 [23.5%]
C-reactive protein, mg/L	14.3 [2.8, 22.2]	8.4 [3.5, 32.3]	8.2 [1.8, 15.7]
Faecal calprotectin, µg/g	412 [36, 3111]	476 [128, 3216]	1325 [211, 1551]
Faecal lactoferrin, µg/g	45.7 [1.3, 221.3]	54.5 [26.6, 198.6]	81.8 [14.8, 173.7]
Prior failure of antibiotics for perianal fistulas	8 [53.3%]	12 [48.0%]	9 [52.9%]
Prior failure of immunomodulators for perianal fistulas	6 [40.0%]	19 [76.0%]	12 [70.6%]
Number of prior biologics used ≥3	4 [26.7%]	12 [48.0%]	6 [35.3%]
Prior failure of TNF inhibitor therapy for perianal fistulas	9 [60.0%]	16 [64.0%]	12 [70.6%]
Prior failure of vedolizumab	2 [13.3%]	4 [16.0%]	5 [29.4%]
Prior failure of ustekinumab	3 [20.0%]	10 [40.0%]	3 [17.6%]
Concomitant use of vedolizumab	1 [6.7%]	2 [8.0%]	1 [5.9%]
Concomitant use of antibiotics for the treatment of PFCD	3 [20.0%]	4 [16.0%]	4 [23.5%]
Concomitant use of systemic corticosteroids and/or immunomodulators	5 [33.3%]	9 [36.0%]	5 [29.4%]

Data are mean [SD], *n* [%], or median [IQR]. CDAI, Crohn’s Disease Activity Index; EO, external opening; IQR, interquartile range; MRI, magnetic resonance imaging; PDAI, Perianal Crohn’s Disease Activity Index; PFCD, perianal fistulising Crohn’s disease; SD, standard deviation; SES-CD, Simple Endoscopic Score for CD; TNF, tumour necrosis factor.

^a^Active luminal disease: CDAI score ≥150.

^b^Complex perianal fistulas on MRI: multiple simple fistulas or single branched [multiple EOs arising from one fistula tract], trans-, extra-, or supra-sphincteric fistulas tracts, possible extensions, and/or fluid collections associated with the perianal fistulas.

**Figure 2.**
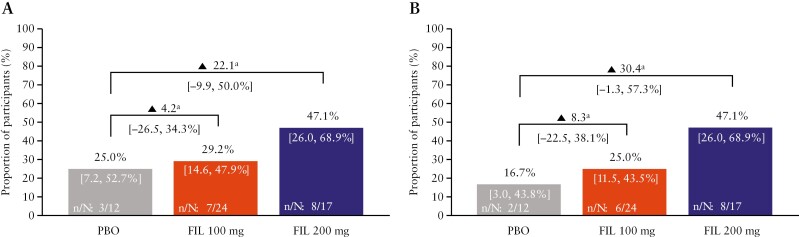
Combined fistula response and combined fistula remission at Week 24 among participants who had at least one draining EO at baseline [subset of the full analysis set]. [A] Proportion of participants who achieved a combined fistula response [reduction of at least one from baseline in the number of draining EOs and an absence of fluid collections of >1 cm on MRI]. [B] Proportion of participants who achieved combined fistula remission [closure of all EOs that were draining at baseline]. Values in parentheses are 90% CI. ^a^Risk difference in proportions: non-responder imputation. CI, confidence interval; EO, external opening; FIL, filgotinib; MRI, magnetic resonance imaging; PBO, placebo.

The proportion of participants achieving the primary endpoint [combined fistula response at Week 24] was numerically higher in the filgotinib 200 mg group (47.1% [8/17]; 90% CI 26.0, 68.9%) than in the placebo group (25.0% [3/12]; 90% CI 7.2, 52.7%) [Figure 2A]. In the filgotinib 100 mg group, 29.2% [7/24; 90% CI 14.6, 47.9%] of participants achieved a combined fistula response at Week 24. Of those participants found not to have met the primary endpoint, three of nine participants in the filgotinib 200 mg group, 13 of 17 participants in the filgotinib 100 mg group, and seven of nine participants in the placebo group did not meet the endpoint based on missing data [Supplementary Table 7]. Similar results were observed for combined fistula remission at Week 24, with a numerical difference seen in favour of filgotinib 200 mg compared with placebo (47.1% [8/17]; 90% CI 26.0, 68.9% versus 16.7% [2/12]; 90% CI 3.0, 43.8%) [Figure 2B]. In the filgotinib 100 mg group, 25.0% [6/24; 90% CI 11.5, 43.5%] of participants achieved combined fistula remission at Week 24. Of those participants who did not achieve combined fistula remission, four of nine participants in the filgotinib 200 mg group, 13 of 17 participants in the filgotinib 100 mg group, and seven of nine participants in the placebo group did not meet the endpoint based on missing data [Supplementary Table 7].

**Figure 3.**
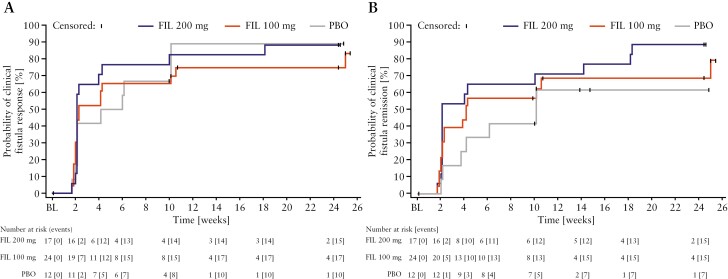
Kaplan–Meier curves of time-to-event endpoints among participants who had at least one draining EO at baseline [subset of the full analysis set]. [A] Time to clinical fistula response [reduction of at least one from baseline in the number of draining EOs that were present at baseline]. [B] Time to clinical fistula remission [closure of all EOs that were draining at baseline]. EO, external opening; FIL, filgotinib; PBO, placebo.

The median time to a clinical fistula response was numerically shorter in the filgotinib 200 mg [15.0 days; 90% CI 15.0, 28.0] and filgotinib 100 mg [16.0 days; 90% CI 15.0, 71.0] groups than in the placebo group [35.5 days; 90% CI 15.0, 71.0] [Figure 3A]. Similarly, the median time to clinical fistula remission was numerically shorter in the filgotinib 200 mg [15.0 days; 90% CI 15.0, 70.0] and filgotinib 100 mg [29.0 days; 90% CI 16.0, 74.0] groups than in the placebo group [71.0 days; 90% CI 26.0, higher bound not available] [Figure 3B].

The proportion of participants achieving proctitis remission at Week 24 [assessed only in the subgroup with moderately to severely active proctitis at baseline] was 10.0% [1/10; 90% CI 0.5, 39.4%] in the filgotinib 200 mg group, 15.4% [2/13; 90% CI 2.8, 41.0%] in the filgotinib 100 mg group, and 28.6% [2/7; 90% CI 5.3, 65.9%] in the placebo group [Supplementary Figure 1]. Identical results were observed for the proportions of participants with a ≥50% reduction in proctitis SES-CD score at Week 24 [Supplementary Figure 2]. Of those participants who did not achieve proctitis remission or a ≥50% reduction in proctitis SES-CD score at Week 24, one of nine participants in the filgotinib 200 mg group, nine of 11 participants in the filgotinib 100 mg group, and three of five participants in the placebo group did not meet the endpoint based on missing data [Supplementary Table 7].

Numerically greater reductions from baseline to Week 10 in mean CDAI scores were observed with filgotinib 200 mg than with placebo, with mean [SD] changes of −69 [76.9] and − 13 [120.4], respectively [Supplementary Table 3]. Mean [SD] changes in CDAI scores from baseline to Week 24 [assessed only in the small number of remaining participants who had moderately active luminal CD at baseline] were − 123 [85.2] [*n *= 6], −39 [17.7] [*n* = 2], and − 138 [*n *= 1] for the filgotinib 200 mg, filgotinib 100 mg, and placebo groups, respectively.

**Figure 4.**
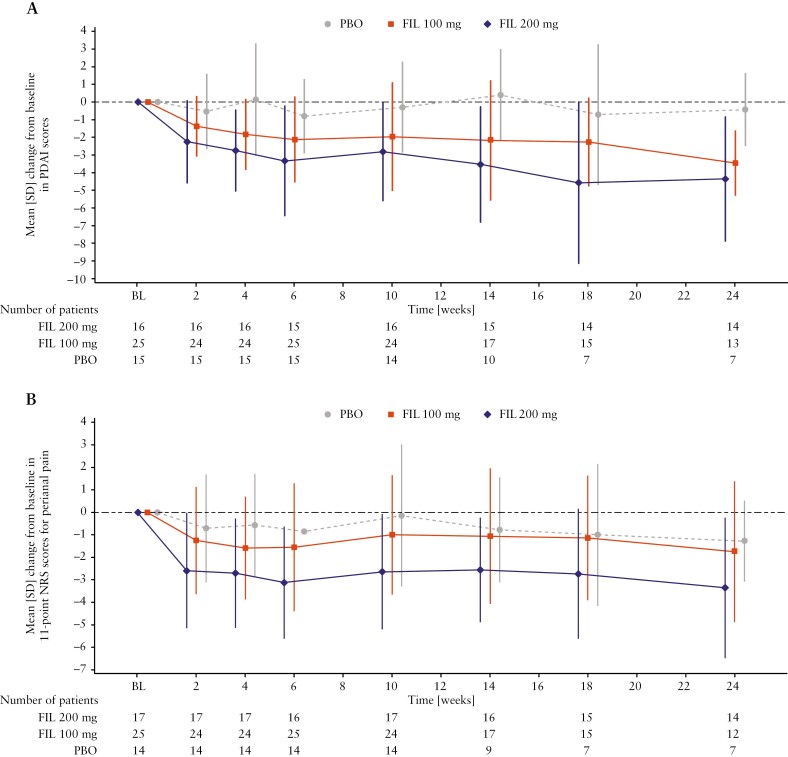
PDAI scores and 11-point NRS scores for perianal pain [full analysis set]. [A] Mean [SD] change from baseline in PDAI scores. [B] Mean [SD] change from baseline in 11-point NRS scores for perianal pain. BL, baseline; FIL, filgotinib; NRS, numerical rating scale; PBO, placebo; PDAI, Perianal Crohn’s Disease Activity Index; SD, standard deviation.

Mean [SD] baseline PDAI scores were 8.6 [3.1] in the filgotinib 200 mg group, 8.3 [3.4] in the filgotinib 100 mg group, and 6.6 [3.1] in the placebo group. Mean [SD] changes from baseline to Week 24 in PDAI scores were numerically greater with filgotinib 200 mg (−4.4 [3.5]) and filgotinib 100 mg (−3.5 [1.9]) than with placebo (−0.4 [2.1]) [Figure 4A]. The mean [SD] change from baseline in 11-point NRS perianal pain score at Week 24 was numerically greater in the filgotinib 200 mg group (−3.4 [3.1]) than the placebo group (−1.3 [1.8]) [Figure 4B]; the mean [SD] change from baseline was − 1.8 [3.1] with filgotinib 100 mg. Numerical trends towards greater reductions in PDAI scores and 11-point NRS perianal pain scores with filgotinib 200 mg compared with placebo were observed as early as Week 2 and were sustained throughout the trial [Figure 4].

There was a trend in favour of filgotinib 200 mg compared with placebo for reductions in CRP levels from baseline to Week 10 [Supplementary Table 4], with a median [interquartile range] change of − 1.3 mg/L [−7.5, 0.6] for the filgotinib 200 mg group and − 0.1 mg/L [−6.4, 2.4] for the placebo group. There were no clear trends for differences in changes from baseline to Week 24 in faecal calprotectin or lactoferrin levels between filgotinib- and placebo-treated groups [Supplementary Tables 5 and 6].

The mean [SD] total durations of exposure to study drug were 22.9 [3.7] weeks, 19.5 [7.5] weeks, and 18.3 [6.6] weeks in the filgotinib 200 mg, filgotinib 100 mg, and placebo groups, respectively. No deaths were reported during the study. Treatment-emergent serious AEs were highest in the filgotinib 200 mg group [Table 2]. TEAE rates were otherwise similar across treatment groups. Serious infections were reported in 2/17 [11.8%] participants in the filgotinib 200 mg group [one participant reported severe bronchitis and another reported suspected COVID-19], 0/25 participants in the filgotinib 100 mg group, and 1/15 [6.7%] participants in the placebo group [a vulval abscess]. There were no reports of herpes zoster, opportunistic infections, malignancies, gastrointestinal perforation, MACE, or thromboembolic events in any of the treatment groups. The most common TEAE was worsening CD (3/17 [17.6%] for filgotinib 200 mg, 4/25 [16.0%] for filgotinib 100 mg, and 1/15 [6.7%] for placebo). The only grade 3 or 4 laboratory abnormality [not treatment-related] reported for at least two participants in a treatment group was grade 3 hypophosphataemia in the filgotinib 100 mg group (5/24 [20.8%]). Only one grade 4 laboratory abnormality [not treatment-related] was reported across all treatment groups: an increase in creatine kinase levels in 1/24 participants [4.2%] was observed in the filgotinib 100 mg group. There were no clinically relevant changes in vital signs or clinically significant electrocardiogram abnormalities in any of the treatment groups. In addition, no treatment-related serious AEs were reported.

**Table 2:** Treatment-emergent adverse events [safety analysis set].

	Placebo [*n* = 15]	Filgotinib 100 mg [*n* = 25]	Filgotinib 200 mg [*n *= 17]
Any TEAE	11 [73.3%]	18 [72.0%]	14 [82.4%]
TEAE grade 3 or higher^a^	0	4 [16.0%]	6 [35.3%]
TEAE related to study drug	2 [13.3%]	2 [8.0%]	4 [23.5%]
TESAE	1 [6.7%]	2 [8.0%]	5 [29.4%]
TESAE related to study drug	0	0	0
Death	0	0	0
TEAE of interest			
Infections	8 [53.3%]	9 [36.0%]	11 [64.7%]
Serious infections	1^b^ [6.7%]	0	2^c^ [11.8%]
Opportunistic infections	0	0	0
Herpes zoster	0	0	0
Malignancies [excluding NMSC]	0	0	0
NMSC	0	0	0
Gastrointestinal perforation	0	0	0
MACE	0	0	0
Thromboembolic events			
Arterial systemic thromboembolism	0	0	0
Venous thromboembolism	0	0	0
TEAEs occurring in ≥2 participants in any treatment group			
Anal abscess	1 [6.7%]	0	3 [17.6%]
Anal fistula	2 [13.3%]	2 [8.0%]	2 [11.8%]
Arthralgia	0	2 [8.0%]	3 [17.6%]
Worsening Crohn’s disease	1 [6.7%]	4 [16.0%]	3 [17.6%]
Fatigue	0	3 [12.0%]	3 [17.6%]
Influenza	2 [13.3%]	1 [4.0%]	3 [17.6%]
Nasopharyngitis	2 [13.3%]	2 [8.0%]	2 [11.8%]
Nausea	1 [6.7%]	2 [8.0%]	2 [11.8%]
Oral herpes	1 [6.7%]	0	2 [11.8%]
Oropharyngeal pain	1 [6.7%]	2 [8.0%]	1 [5.9%]
Palpitations	0	2 [8.0%]	0
Rash	2 [13.3%]	1 [4.0%]	1 [5.9%]

Data are *n* [%]. A TEAE was defined as any AE that began on or after the day of the first dose up to 30 days after the last dose, or that led to discontinuation of study drug. Multiple AEs were counted only once per participant for each preferred term.

COVID-19, Coronavirus disease 2019; MACE, major adverse cardiovascular event; NMSC, nonmelanoma skin cancer; TEAE, treatment-emergent adverse event; TESAE, treatment-emergent serious adverse event.

^a^Severity grades were defined by the Common Terminology Criteria for Adverse Events.

^b^One participant reported a vulval abscess.

^c^One participant reported severe bronchitis, one participant reported suspected COVID-19.

## 4. Discussion

In this phase 2, proof-of-concept study in adults with PFCD and prior treatment failure, treatment with filgotinib 200 mg for 24 weeks led to numerical reductions compared with placebo in the number of draining perianal fistulas, as determined by physical assessment combined with the absence of fluid collections of >1 cm in at least two dimensions on pelvic MRI. Treatment with filgotinib 200 mg for 24 weeks also improved the severity of perianal disease compared with placebo, as determined by PDAI and 11-point NRS perianal pain scores. Treatment effects were not as pronounced, or were absent, with filgotinib 100 mg. Filgotinib 100 mg and filgotinib 200 mg were generally well tolerated.

This is the first randomised, placebo-controlled trial to evaluate the efficacy and safety of a JAK inhibitor in participants with PFCD. Three pivotal trials have previously assessed potential treatments for perianal fistulas: one for the TNF inhibitor infliximab,^6^ one for the calcineurin inhibitor tacrolimus,^17^ and one for the human allogeneic mesenchymal stem cell therapy darvadstrocel.^8^ A pertinent difference between the primary endpoints of these trials and the DIVERGENCE 2 study was the MRI assessment component of the composite endpoint in DIVERGENCE 2, which evaluated an absence of fluid collections of >1 cm on pelvic MRI. Assessments of infliximab and tacrolimus efficacy were based only on physical evaluations of the participants,^5,17^ and although in the darvadstrocel trial the clinical assessment component of the composite endpoint [ie, closure of all treated EOs that were draining at baseline] was more stringent than that in DIVERGENCE 2, the criterion for the MRI assessment component was an absence of fluid collections of >2 cm.^8^ Notably, in DIVERGENCE 2, all participants in the filgotinib 200 mg group who achieved the primary endpoint of combined fistula response at Week 24 also achieved combined fistula remission [ie, closure of all draining EOs that were present at baseline] at Week 24; the placebo effect at Week 24 for combined fistula remission was smaller than for combined fistula response. Thus, there was a greater numerical difference in the proportions of participants who achieved combined fistula remission than combined fistula response between the filgotinib 200 mg and placebo groups. It is also noteworthy that more than 50% of participants in DIVERGENCE 2 had moderately to severely active proctitis at baseline, a potential negative predictor for fistula healing.^18–20^

In this study, the relatively high rates of missing data were largely caused by discontinuations; non-responders [based on CDAI and PDAI scores] at Week 10, and those with worsening luminal disease [based on CDAI scores] from Week 10 to Week 24, were discontinued. This reduced the study’s sample size by 28% and resulted in a greater proportion of placebo- than filgotinib 200 mg-treated participants discontinuing [40% versus 12%, respectively], which may be reflective of treatment benefits of filgotinib. It also created a selection bias for responders after Week 10, which probably affected several endpoints, including proctitis remission and changes from baseline in CDAI scores, PDAI scores, 11-point NRS scores for perianal pain, and inflammatory biomarkers.

In this study, the overall numbers of participants achieving proctitis remission were very low [one in the filgotinib 200 mg group and two each in the filgotinib 100 mg and placebo groups]. To ensure this result was not due to the stringency of the endpoint, the proportion of participants with a ≥50% reduction in proctitis SES-CD score was also assessed in a post hoc analysis. Results of this post hoc analysis were identical to those for the original endpoint. Given the overall very low numbers of participants who achieved proctitis remission, it is possible that the greater number of participants achieving proctitis remission in the placebo group was due to random chance alone. It is notable that this study selected for a clinical fistula response; however, the rate of a proctitis response was very low. Follow-up analyses should focus on assessing whether there is an association between a clinical fistula response and a proctitis response, or between an MRI fistula response and a proctitis response.

Despite the selection bias for responders and a lower mean baseline PDAI score in the placebo group than in the filgotinib groups, there were greater reductions from baseline to Week 24 in PDAI and 11-point NRS perianal pain scores in the filgotinib 200 mg group than in the placebo group. Mean CDAI scores, like median inflammatory biomarker levels, after Week 10 probably reflect the selection bias for responders. In contrast, mean changes from baseline to Week 10 in CDAI scores and CRP levels were consistent with results for other efficacy endpoints at Week 10. For changes in CDAI scores [assessed only in participants who had moderately active luminal CD at baseline], the small number of participants remaining at Week 24 [six, two, and one for filgotinib 200 mg, filgotinib 100 mg, and placebo, respectively] renders any sensible interpretation of these data untenable and, in addition to the selection bias, highlights the difficulty of studying and treating luminal and fistulising disease simultaneously.

In this study, filgotinib had an acceptable safety and tolerability profile, with no new safety signals being observed. Overall, a higher proportion of participants experienced TEAEs of worsening CD in the filgotinib-treated groups than in the placebo group. This may partially be explained by a ‘treat-straight-through’ design, because participants who were able to remain in the study for up to 24 weeks, despite being on placebo, may have had less severely active CD than filgotinib-treated participants. Additionally, given that more participants treated with filgotinib continued past Week 10, the total duration on study drug was longer in the filgotinib-treated groups than in the placebo group, possibly increasing the likelihood that a participant would experience a TEAE during the study by chance alone. It is important to note that the mean total durations of exposure to filgotinib in DIVERGENCE 2 were short [approximately 20 weeks].

A main limitation of the study is that non-responders at Week 10 or participants with worsening of luminal disease symptoms from Week 10 to Week 24 were discontinued. This created a selection bias for responders, notably reducing the number of participants in the filgotinib 100 mg and placebo groups after Week 10. As the participants remaining in the placebo group had experienced a placebo effect and may have had less severe disease than those who were discontinued, the threshold for an observable treatment effect was raised. Another limitation of this study is the small sample size, which warrants cautious interpretation of the findings. In addition to the exploratory study design, DIVERGENCE 2 was unable to reach its recruitment target owing to the COVID-19 pandemic, which led to smaller than anticipated participant numbers, in particular in the filgotinib 200 mg group. A further limitation of this study was that the potential impact of concomitant inflammatory bowel disease medications on the outcomes could not be interrogated owing to the small size of the subgroups; however, concomitant treatment with these medications was balanced between the filgotinib 200 mg and placebo groups.

The question remains as to why potential benefits were seen in this study for the composite endpoints of clinical and MRI assessments, but were less pronounced or absent for most other endpoints. One reason might be the stringency of these endpoints, which was driven primarily by objective MRI findings. Thus, although the study design resulted in the selection of clinical responders after Week 10, including from the placebo group, the clinical response of placebo-treated participants may not have been supported by MRI findings at Week 24, thus allowing greater resolution of the treatment effects of filgotinib compared with placebo for these composite versus other endpoints.

In conclusion, in a population with PFCD and prior treatment failure, treatment with filgotinib 200 mg led to numerical reductions compared with placebo in the number of draining perianal fistulas, as determined by investigator and MRI assessments, and in the severity of perianal disease, as assessed by PDAI and 11-point NRS perianal pain scores. Treatment with filgotinib was generally well tolerated. Overall, data from this exploratory clinical trial suggest that further studies of filgotinib for the treatment of PFCD are warranted.

## Supplementary Data

Supplementary data are available at *ECCO-JCC* online.

jjae003_suppl_Supplementary_Material

## Data Availability

Gilead Sciences shares anonymised individual patient data upon request or as required by law or regulation with qualified external researchers based on submitted curriculum vitae and reflecting non-conflict of interest. The request proposal must also include a statistician. Approval of such requests is at Gilead Science’s discretion and is dependent on the nature of the request, the merit of the research proposed, the availability of the data, and the intended use of the data. Data requests should be sent to [datarequest@gilead.com].

## References

[1] Roda G , Chien Ng S, Kotze PG, et al. Crohn’s disease. Nat Rev Dis Primers 2020;6:22. 32242028 10.1038/s41572-020-0156-2

[2] Sica GS , Di Carlo S, Tema G, et al. Treatment of peri-anal fistula in Crohn’s disease. World J Gastroenterol 2014;20:13205–10. 25309057 10.3748/wjg.v20.i37.13205 PMC4188878

[3] Sandborn WJ , Fazio VW, Feagan BG, Hanauer SB; American Gastroenterological Association Clinical Practice Committee. AGA technical review on perianal Crohn’s disease. Gastroenterology 2003;125:1508–30. 14598268 10.1016/j.gastro.2003.08.025

[4] Lopez N , Ramamoorthy S, Sandborn WJ. Recent advances in the management of perianal fistulising Crohn’s disease: lessons for the clinic. Expert Rev Gastroenterol Hepatol 2019;13:563–77. 31023087 10.1080/17474124.2019.1608818 PMC6545251

[5] Present DH , Rutgeerts P, Targan S, et al. Infliximab for the treatment of fistulas in patients with Crohn’s disease. N Engl J Med 1999;340:1398–405. 10228190 10.1056/NEJM199905063401804

[6] Sands BE , Anderson FH, Bernstein CN, et al. Infliximab maintenance therapy for fistulising Crohn’s disease. N Engl J Med 2004;350:876–85. 14985485 10.1056/NEJMoa030815

[7] Meima-van Praag EM , van Rijn KL, Wasmann KATGM, et al. Short-term anti-TNF therapy with surgical closure versus anti-TNF therapy in the treatment of perianal fistulas in Crohn’s disease [PISA-II]: a patient preference randomised trial. Lancet Gastroenterol Hepatol 2022;7:617–26. 35427495 10.1016/S2468-1253(22)00088-7

[8] Panés J , García-Olmo D, Van Assche G, et al.; ADMIRE CD Study Group Collaborators. Expanded allogeneic adipose-derived mesenchymal stem cells [Cx601] for complex perianal fistulas in Crohn’s disease: a phase 3 randomised, double-blind controlled trial. Lancet 2016;388:1281–90. 27477896 10.1016/S0140-6736(16)31203-X

[9] Panés J , García-Olmo D, Van Assche G, et al; ADMIRE CD Study Group Collaborators. Long-term efficacy and safety of stem cell therapy [Cx601] for complex perianal fistulas in patients with Crohn’s disease. Gastroenterology 2018;154:1334–42.e4. 29277560 10.1053/j.gastro.2017.12.020

[10] European Medicines Agency. Takeda UK. Summary of Product Characteristics, Alofisel. 2023. https://www.ema.europa.eu/en/medicines/human/EPAR/alofisel#product-information-section Accessed March 01, 2023.

[11] Lin CM , Cooles FA, Isaacs JD. Basic mechanisms of JAK inhibition. Mediterr J Rheumatol 2020;31:100–4. 32676567 10.31138/mjr.31.1.100 PMC7361186

[12] Choy EH. Clinical significance of Janus kinase inhibitor selectivity. Rheumatology 2018;58:1122. 10.1093/rheumatology/kez002 PMC6532441 30698756

[13] Danese S , Argollo M, Le Berre C, Peyrin-Biroulet L. Jak selectivity for inflammatory bowel disease treatment: does it clinically matter? Gut 2019;68:1893–9. 31227590 10.1136/gutjnl-2019-318448

[14] European Medicines Agency. Galapagos nv. Summary of Product Characteristics, Jyseleca. 2022. https://www.medicines.org.uk/emc/product/11809/smpc Accessed March 01, 2023.

[15] Galapagos nv. *Jyseleca Approved in Japan for Ulcerative Colitis*. 2022. https://ml-eu.globenewswire.com/Resource/Download/02f3168f-a014-46ed-b06b-06c9b0b1d011 Accessed March 01, 2023.

[16] Vermeire S , Schreiber S, Petryka R, et al. Clinical remission in patients with moderate-to-severe Crohn’s disease treated with filgotinib [the FITZROY study]: results from a phase 2, double-blind, randomised, placebo-controlled trial. Lancet 2017;389:266–75. 27988142 10.1016/S0140-6736(16)32537-5

[17] Sandborn WJ , Present DH, Isaacs KL, et al. Tacrolimus for the treatment of fistulas in patients with Crohn’s disease: a randomised, placebo-controlled trial. Gastroenterology 2003;125:380–8. 12891539 10.1016/s0016-5085(03)00877-1

[18] Schwartz DA , Herdman CR. The medical treatment of Crohn’s perianal fistulas. Aliment Pharmacol Ther 2004;19:953–67. 15113362 10.1111/j.1365-2036.2004.01917.x

[19] Tougeron D , Savoye G, Savoye-Collet C, Koning E, Michot F, Lerebours E. Predicting factors of fistula healing and clinical remission after infliximab-based combined therapy for perianal fistulising Crohn’s disease. Dig Dis Sci 2009;54:1746–52. 19003531 10.1007/s10620-008-0545-y

[20] Brochard C , Rabilloud M-L, Hamonic S, et al. Natural history of perianal Crohn’s disease: long-term follow-up of a population-based cohort. Clin Gastroenterol Hepatol 2022;20:e102–10. 33359730 10.1016/j.cgh.2020.12.024

