# Supplementary material for: Efficacy and Safety of Filgotinib for the Treatment of Perianal Fistulising Crohn’s Disease [DIVERGENCE 2]: A Phase 2, Randomised, Placebo-controlled Trial
Source: J Crohns Colitis. 2024 Feb 16;18(6):864–74. doi: 10.1093/ecco-jcc/jjae003 (PMC11147792; doi:10.1093/ecco-jcc/jjae003)
Supplement: jjae003_suppl_Supplementary_Material [file jjae003_suppl_supplementary_material.docx]

# Supplementary material

# Contents

## Supplementary Table 1. Schedule of assessments.

## Supplementary Table 2. Safety adjudication committee members.

## Supplementary Table 3. Change in CDAI scores over time among participants who had a CDAI score of ≥220 at baseline [full analysis set].

## Supplementary Table 4. Change in serum C-reactive protein [mg/L] over time [biomarker analysis set].

## Supplementary Table 5. Change in faecal calprotectin [µg/g] from baseline to week 24 [biomarker analysis set].

## Supplementary Table 6. Change in faecal lactoferrin [µg/g] from baseline to week 24 [biomarker analysis set].

## Supplementary Table 7. Rates of missing data for binary endpoints

## Supplementary Figure 1. Proctitis remission at week 24 [full analysis set].

## Supplementary Figure 2. Reduction of ≥50% in proctitis SES-CD score at week 24 [full analysis set].

**Supplementary Table 1.** Schedule of assessments.

|  | **Screening** | **Treatment** | | | | | | | **Follow-up** | | | |
| --- | --- | --- | --- | --- | --- | --- | --- | --- | --- | --- | --- | --- |
| **Visit** | **1** | **2** | **3** | **4** | **5** | **6** | **7** | **8** | **9** | **10** | **PTx^a^** | **ET** |
| **Week** | ·· | **0** | **2** | **4** | **6** | **10** | **14** | **18** | **20** | **24** | ·· | ·· |
| **Study day** | **–30 to –1** | **1** | **15** | **29** | **43** | **71** | **99** | **127** | **141** | **169** | ·· | ·· |
| **Visit window** | ·· | ·· | **±3** | **±3** | **±3** | **±2** | **±3** | **±3** | **±5** | **±5** | **±3** | ·· |
| Written informed consent | X | ·· | ·· | ·· | ·· | ·· | ·· | ·· | ·· | ·· | ·· | ·· |
| Medical history and demographics | X | ·· | ·· | ·· | ·· | ·· | ·· | ·· | ·· | ·· | ·· | ·· |
| Crohn’s disease and treatment history | X | ·· | ·· | ·· | ·· | ·· | ·· | ·· | ·· | ·· | ·· |  |
| 12-lead ECG | X | ·· | ·· | ·· | ·· | X | ·· | ·· | ·· | ·· | ·· | X^b^ |
| Review of inclusion/exclusion criteria | X | X | ·· | ·· | ·· | ·· | ·· | ·· | ·· | ·· | ·· | ·· |
| PE [complete including perianal]^c^ | X | ·· | ·· | ·· | ·· | ·· | ·· | ·· | ·· | ·· | ·· | ·· |
| PE [symptom-based] and perianal assessment^c^ | ·· | X | X | X | X | X | X | X | ·· | X | X | X |
| Vital signs | X | X | X | X | X | X | X | X | ·· | X | X | X |
| Weight | X | X | X | X | X | X | X | X | ·· | X | X | X |
| Height | X | ·· | ·· | ·· | ·· | ·· | ·· | v | ·· | ·· | ·· | ·· |
| Adverse events | X | X | X | X | X | X | X | X | ·· | X | X | X |
| Concomitant medications | X | X | X | X | X | X | X | X | ·· | X | X | X |
| Randomisation | ·· | X | ·· | ·· | ·· | ·· | ·· | ·· | ·· | ·· | ·· | ·· |
| Study drug dispensing | ·· | X | ·· | X | ·· | X | X | X | ·· | ·· | ·· | ·· |
| CDAI^d^ | X | ·· | X | X | X | X | X | X | ·· | X | ·· | X |
| PRO2^d^ | X | ·· | X | X | X | X | X | X | ·· | X | ·· | X |
| PDAI | ·· | X | X | X | X | X | X | X | ·· | X | ·· | X |
| 11-point NRS score for perianal pain | X | X | X | X | X | X | X | X | ·· | X | ·· | X |
| eDiary instruction and review^e^ | X | X | X | X | X | X | X | X | ·· | X | ·· | ·· |
| Flexible sigmoidoscopy | X | ·· | ·· | ·· | ·· | ·· | ·· | ·· | ·· | X | ·· | X^f^ |
| Pelvic MRI | X | ·· | ·· | ·· | ·· | ·· | ·· | ·· | ·· | X | ·· | X^g^ |
| Stool for *C. diff* toxin, pathogenic *E. coli*, *Salmonella*, *Shigella*, *Campylobacter*, and *Yersinia* testing | X | ·· | ·· | ·· | ·· | ·· | ·· | ·· | ·· | ·· | ·· | ·· |
| Stool O&P | X | ·· | ·· | ·· | ·· | ·· | ·· | ·· | ·· | ·· | ·· | ·· |
| Stool microbiome | X | ·· | ·· | ·· | ·· | ·· | ·· | ·· | ·· | X | ·· | ·· |
| Faecal biomarkers | X | ·· | ·· | ·· | ·· | ·· | ·· | ·· | ·· | X | ·· | ·· |
| Faecal lactoferrin and calprotectin | X | ·· | ·· | ·· | ·· | ·· | ·· | ·· | ·· | X | ·· | ·· |
| Urine drug screen^h^ | X | ·· | ·· | ·· | ·· | ·· | ·· | ·· | ·· | ·· | ·· | ·· |
| Urinalysis | X | ·· | ·· | ·· | ·· | ·· | ·· | ·· | ·· | X | ·· | ·· |
| Pregnancy test^i^ | X | X | ·· | X | X | X | X | X | X | X | X | X |
| TB screening^j^ | X | ·· | ·· | ·· | ·· | ·· | ·· | ·· | ·· | ·· | ·· | ·· |
| Chest x-ray^k^ | X | ·· | ·· | ·· | ·· | ·· | ·· | ·· | ·· | ·· | ·· | ·· |
| HBV, HCV, and HIV screening^l^ | X | ·· | ·· | ·· | ·· | ·· | ·· | ·· | ·· | ·· | ·· | ·· |
| Haematology | X | X | X | X | X | X | X | X | ·· | X | X | X |
| Chemistry | X | X | X | X | X | X | X | X | ·· | X | X | X |
| Fasting lipids^m^ | ·· | X | ·· | ·· | ·· | X | ·· | ·· | ·· | X | ·· | ·· |
| CRP | X | X | X | X | X | X | X | X | ·· | X | X | X |
| Blood TCR/BCR repertoire sample | ·· | X | ·· | X | ·· | X | ·· | ·· | ·· | X | ·· | ·· |
| Plasma biomarker sample | ·· | X | ·· | X | ·· | X | ·· | ·· | ·· | X | ·· | ·· |
| Serum biomarker sample | ·· | X | ·· | X | ·· | X | ·· | ·· | ·· | X | ·· | ·· |
| Blood transcriptome sample | ·· | X | ·· | X | ·· | X | ·· | ·· | ·· | X | ·· | ·· |
| vfPBMC | ·· | X | ·· | X | ·· | X | ·· | ·· | ·· | X | ·· | ·· |
| Serum immunoglobulin | ·· | X | ·· | X | ·· | X | ·· | ·· | ·· | X | X | X |
| PK sampling [sparse]^n^ | ·· | ·· | ·· | X | ·· | X | ·· | X | ·· | X | ·· | ·· |
| Genomic sub-study [optional]^o^ | ·· | X | ·· | ·· | ·· | ·· | ·· | ·· | ·· | ·· | ·· | ·· |
| HRQoL questionnaires^p^ | ·· | X | ·· | ·· | ·· | X | ·· | ·· | ·· | X | ·· | ·· |

BCR, B-cell receptor; CDAI, Crohn's Disease Activity Index; *C*. *diff*, *Clostridium difficile*; CRP, C-reactive protein; ECG, electrocardiogram; *E*. *coli*, *Escherichia coli*; eCRF, electronic case report form; ET, early termination; HBV, hepatitis B virus; HCV, hepatitis C virus; HIV, human immunodeficiency virus; HRQoL, health-related quality of life; IBDQ, Inflammatory Bowel Disease Questionnaire; LTE, long-term extension; MRI, magnetic resonance imaging; NRS, numeric rating scale; O&P, ova and parasites; PDAI, Perianal Crohn’s Disease Activity Index; PE, physical examination; PK, pharmacokinetic; PRO2, patient-reported outcome consisting of two items; PTx, post treatment; SF-36, 36-Item Short Form Survey; TB, tuberculosis; TCR, T-cell receptor; vfPBMC, viably frozen peripheral blood mononuclear cell; WPAI, Work Productivity and Activity Impairment Questionnaire.

^a^The PTx visit occurred 30 days after the last dose of study drug. Only participants who rolled over into the LTE study did not complete PTx assessments.

^b^For participants who terminated before week 10.

^c^A complete PE including vital signs, body weight, height, and perianal assessment [including completion of the Perianal Fistula Assessment Worksheet] was performed at

screening. A symptom-directed PE and perianal assessment [including completion of the Perianal Fistula Assessment Worksheet] was performed at all other time points.

^d^The screening CDAI score was used as the day 1 measurement. The CDAI participant-reported outcomes of stool frequency and abdominal pain were used to derive the PRO2 score.

^e^Participants began filling in the eDiary on the day of their initial screening visit and continued to fill it in throughout the remainder of the study.

^f^Participants meeting non-response or disease worsening criteria underwent a flexible sigmoidoscopy assessment before exiting the study.

^g^Participants meeting non-response or disease worsening criteria underwent a pelvic MRI examination before exiting the study.
^h^A positive cocaine test disqualified participants; positive amphetamine, barbiturate, benzodiazepine, and opioid tests required medical monitor review.

^i^All women meeting the childbearing potential criteria had a serum pregnancy test at screening, and a urine pregnancy test was completed at least every 4 weeks. If any pregnancy test was positive, study drug was immediately interrupted, and the participant came to the site for a serum pregnancy test in the clinic.

^j^Proof of no active or untreated latent TB at screening. Participants who were diagnosed with latent TB at screening initiated an adequate course of prophylaxis as per local

standard of care, for a minimum of 4 weeks before randomisation. Participants initiated study drug dosing only after consultation with the study medical monitor.

^k^Chest x-rays [views as per local guidelines] were performed at screening or in the 3 months before screening [with the report or films available for investigator review] without evidence of active or latent TB infection.

^l^An HIV-1/HIV-2 antibody test, an HCV antibody test, an HBV surface antigen test, an HBV surface antibody test, and an HBV core antibody test were performed in all participants. Participants with a positive HBV core antibody test required reflex testing for HBV DNA [conducted locally].

^m^Participants fasted [no food or drink, except water] for at least 8 hours before blood sample collection on day 1, at week 10, and at week 24.

^n^Sparse PK sampling was collected before dosing at week 10 and week 24 [in the 2 hours before dosing], after dosing at week 4 [at least 30 minutes and up to 3 hours after supervised dosing in a clinic], and anytime at week 18. For all visits with PK sampling, the time of the dose taken before and on the day of the visit was noted in the eCRF.

^o^Only for participants who consented to the optional genomic sub-study. The sample was obtained at a subsequent visit if not obtained on day 1.

^p^HRQoL assessments included the SF-36, WPAI, EQ-5D, and IBDQ.

**Supplementary Table 2.** Safety adjudication committee members.

|  | **Title** |
| --- | --- |
| **Robert P Giugliano** | Adjudication Committee Chairman |
| **Megan Leary** | Adjudicator, Neurologist |
| **Duane Pinto** | Adjudicator, Cardiologist |
| **Eli Gelfand** | Adjudicator, Cardiologist |
| **Sarah Hanson** | Program Manager, Clinical Adjudication, Bioclinica |
| **Judith Narisi** | Director, Clinical Adjudication and Eligibility, Bioclinica |
| **Mona Trivedi** | Senior Director, Clinical Research [MD] |
| **Chohee Yun** | Senior Director, Clinical Research [MD] |
| **Lien Gheyle** | Medical Director, Clinical Research [MD] |

**Supplementary Table 3.** Change in CDAI scores over time among participants who had a CDAI score of ≥220 at baseline [full analysis set].

|  | **Placebo** | | | **Filgotinib 100 mg** | | | **Filgotinib 200 mg** | | |
| --- | --- | --- | --- | --- | --- | --- | --- | --- | --- |
|  | **n** | **Mean [SD]** | **Mean [SD]  change from BL** | **n** | **Mean [SD]** | **Mean [SD]  change from BL** | **n** | **Mean [SD]** | **Mean [SD]  change from BL** |
| **BL** | 4 | 258 [30.0] | ·· | 10 | 260 [22.9] | ·· | 7 | 243 [21.5] | ·· |
| **Week 6** | 4 | 220 [98.8] | −38 [100.1] | 8 | 242 [104.2] | −13 [91.1] | 7 | 130 [22.0] | −112 [15.5] |
| **Week 10** | 4 | 246 [124.2] | −13 [120.4] | 9 | 245 [79.1] | −11 [71.4] | 7 | 174 [80.8] | −69 [76.9] |
| **Week 14** | 1 | 183 | −106 | 4 | 237 [151.3] | −13 [145.2] | 3 | 115 [77.4] | −118 [83.7] |
| **Week 18** | 1 | 166 | −123 | 3 | 228 [62.3] | −20 [65.8] | 6 | 145 [82.7] | −91 [83.5] |
| **Week 24** | 1 | 151 | −138 | 2 | 213 [42.4] | −39 [17.7] | 6 | 113 [83.2] | −123 [85.2] |

BL, baseline; CDAI, Crohn’s Disease Activity Index; SD, standard deviation.

**Supplementary Table 4.** Change in serum C-reactive protein [mg/L] over time [biomarker analysis set].

|  | **Placebo** | | | **Filgotinib 100 mg** | | | **Filgotinib 200 mg** | | |
| --- | --- | --- | --- | --- | --- | --- | --- | --- | --- |
|  | **n** | **Median [IQR]** | **Median [IQR]  change from BL** | **n** | **Median [IQR]** | **Median [IQR]  change from BL** | **n** | **Median [IQR]** | **Median [IQR]  change from BL** |
| **BL** | 15 | 14.3 [2.8, 22.2] | ·· | 24 | 7.9 [3.4, 40.4] | ·· | 17 | 8.2 [1.8, 15.7] | ·· |
| **Week 6** | 15 | 9.1 [4.6, 20.8] | −2.5 [−6.2, 3.2] | 22 | 14.4 [2.9, 35.2] | −0.2 [−4.1, 5.3] | 16 | 3.7 [0.5, 9.0] | −3.0 [−6.6, −0.5] |
| **Week 10** | 15 | 9.3 [3.6, 24.2] | −0.1 [−6.4, 2.4] | 24 | 7.3 [2.3, 37.3] | 0.1 [−2.3, 7.5] | 17 | 3.8 [0.6, 16.0] | −1.3 [−7.5, −0.6] |
| **Week 14** | 11 | 14.0 [3.5, 17.0] | −1.1 [−5.4, 3.0] | 18 | 6.2 [2.4, 25.0] | −2.2 [−11.5, 0.1] | 15 | 9.8 [0.7, 18.8] | −0.7 [−7.5, 7.3] |
| **Week 18** | 7 | 7.7 [1.4, 16.9] | −1.6 [−15.9, 5.9] | 15 | 9.8 [2.9, 27.1] | −1.3 [−15.7, 0.6] | 15 | 5.8 [0.7, 20.8] | −1.1 [−9.2, 0.0] |
| **Week 24** | 6 | 10.2 [4.5, 22.6] | −2.0 [−10.3, 1.6] | 13 | 2.7 [1.3, 7.4] | −2.4 [−11.4, −0.8] | 15 | 3.1 [0.7, 17.7] | −0.1 [−7.1, 8.1] |

BL, baseline; IQR, interquartile range.

**Supplementary Table 5.** Change in faecal calprotectin [µg/g] from baseline to week 24 [biomarker analysis set].

|  | **Placebo** | | | **Filgotinib 100 mg** | | | **Filgotinib 200 mg** | | |
| --- | --- | --- | --- | --- | --- | --- | --- | --- | --- |
|  | **n** | **Median [IQR]** | **Median [IQR]  change from BL** | **n** | **Median [IQR]** | **Median [IQR]  change from BL** | **n** | **Median [IQR]** | **Median [IQR]  change from BL** |
| **BL** | 15 | 412 [36, 3111] | ·· | 22 | 430 [128, 3216] | ·· | 17 | 1325 [211, 1551] | ·· |
| **Week 24** | 5 | 76 [67, 582] | 38 [0, 118] | 9 | 363 [73, 1165] | −12 [−166, 195] | 14 | 444 [159, 1091] | −2 [−492, 176] |

BL, baseline; IQR, interquartile range.

**Supplementary Table 6.** Change in faecal lactoferrin [µg/g] from baseline to week 24 [biomarker analysis set].

|  | **Placebo** | | | **Filgotinib 100 mg** | | | **Filgotinib 200 mg** | | |
| --- | --- | --- | --- | --- | --- | --- | --- | --- | --- |
|  | **n** | **Median [IQR]** | **Median [IQR]  change from BL** | **n** | **Median [IQR]** | **Median [IQR]  change from BL** | **n** | **Median [IQR]** | **Median [IQR]  change from BL** |
| **BL** | 15 | 45.7 [1.3, 221.3] | ·· | 24 | 53.6 [23.7, 194.0] | ·· | 17 | 81.8 [14.8, 173.7] | ·· |
| **Week 24** | 5 | 5.5 [0.9, 10.5] | 0.0 [−0.4, 2.8] | 12 | 21.5 [3.4, 57.9] | −4.1 [−29.8, 12.0] | 13 | 64.1 [14.6, 83.1] | −12.4 [−109.6, 49.6] |

BL, baseline; IQR, interquartile range.

**Supplementary Table 7.** Rates of missing data for binary endpoints [full analysis set].

|  | **Placebo**  **n [%]** | **Filgotinib 100 mg n [%]** | **Filgotinib 200 mg**  **n [%]** |
| --- | --- | --- | --- |
| **Primary endpoint: Proportion of participants who achieved combined fistula response at week 24 [among participants with ≥1 draining EO at baseline]** | | | |
| **Participants with ≥1 draining EO at baseline** | 17 | 24 | 12 |
| **Participants who achieved combined fistula response** | 3 [25.0] | 7 [29.2] | 8 [47.1] |
| **Participants who did not achieve combined fistula response** | 9 [75.0] | 17 [70.8] | 9 [52.9] |
| **Not meeting the endpoint based on observed data** | 2 [16.7] | 4 [16.7] | 6 [35.3] |
| **Non-responders due to treatment failure^a^** | 0 | 3 [12.5] | 0 |
| **Protocol-specified CD non-responder at week 10^b^** | 4 [33.3] | 5 [20.8] | 0 |
| **Protocol-specified PFCD non-responder at week 10^c^** | 0 | 0 | 1 [5.9] |
| **Non-responder due to luminal disease worsening^d^** | 3 [25.0] | 1 [4.2] | 1 [5.9] |
| **Insufficient data due to early termination** | 0 | 4 [16.7] | 1 [5.9] |
| **Insufficient data due to other reasons** | 0 | 0 | 0 |
| **Secondary endpoint: Proportion of participants who achieved combined fistula remission at week 24 [among participants with ≥1 draining EO at baseline]** | | | |
| **Participants with ≥1 draining EO at baseline** | 17 | 24 | 12 |
| **Participants who achieved combined fistula remission** | 2 [16.7] | 6 [25.0] | 8 [47.1] |
| **Participants who did not achieve combined fistula remission** | 10 [83.3] | 18 [75.0] | 9 [52.9] |
| **Not meeting the endpoint based on observed data** | 3 [25.0] | 5 [20.8] | 6 [35.3] |
| **Non-responders due to treatment failure^a^** | 0 | 3 [12.5] | 0 |
| **Protocol-specified CD non-responder at week 10^b^** | 4 [33.3] | 5 [20.8] | 0 |
| **Protocol-specified PFCD non-responder at week 10^c^** | 0 | 0 | 1 [5.9] |
| **Non-responder due to luminal disease worsening^d^** | 3 [25.0] | 1 [4.2] | 2 [5.9] |
| **Insufficient data due to early termination** | 0 | 4 [16.7] | 1 [5.9] |
| **Insufficient data due to other reasons** | 0 | 0 | 0 |
| **Secondary endpoint: Proportion of participants who achieved proctitis remission at week 24 [among participants who had moderately to severely active proctitis at baseline]** | | | |
| **Participants who had moderately to severely active proctitis at baseline** | 7 | 13 | 10 |
| **Participants who achieved proctitis remission** | 2 [28.6] | 2 [15.4] | 1 [10.0] |
| **Participants who did not achieve proctitis response** | 5 [71.4] | 11 [84.6] | 9 [90.0] |
| **Not meeting the endpoint based on observed data** | 2 [28.6] | 2 [15.4] | 8 [80.0] |
| **Non-responders due to treatment failure^a^** | 0 | 0 | 0 |
| **Protocol-specified CD non-responder at week 10^b^** | 1 [14.3] | 3 [23.1] | 0 |
| **Protocol-specified PFCD non-responder at week 10^c^** | 0 | 0 | 1 [10.0] |
| **Non-responder due to luminal disease worsening^d^** | 2 [28.6] | 2 [15.4] | 0 |
| **Insufficient data due to early termination** | 0 | 3 [23.1] | 0 |
| **Insufficient data due to other reasons** | 0 | 1 [7.7] | 0 |
| ***Post hoc* analysis: Proportion of participants who achieved ≥50% reduction in proctitis SES-CD scores at week 24 [among participants who had moderately to severely active proctitis at baseline]** | | | |
| **Participants who had moderately to severely active proctitis at baseline** | 7 | 13 | 10 |
| **Participants with ≥50% reduction in proctitis SES-CD scores** | 2 [28.6] | 2 [15.4] | 1 [10.0] |
| **Participants who did not achieve ≥50% reduction in proctitis SES-CD scores** | 5 [71.4] | 11 [84.6] | 9 [90.0] |
| **Not meeting the endpoint based on observed data** | 2 [28.6] | 2 [15.4] | 8 [80.0] |
| **Non-responders due to treatment failure^a^** | 0 | 0 | 0 |
| **Protocol-specified CD non-responder at week 10^b^** | 1 [14.3] | 3 [23.1] | 0 |
| **Protocol-specified PFCD non-responder at week 10^c^** | 0 | 0 | 1 [10.0] |
| **Non-responder due to luminal disease worsening^d^** | 2 [28.6] | 2 [15.4] | 0 |
| **Insufficient data due to early termination** | 0 | 3 [23.1] | 0 |
| **Insufficient data due to other reasons** | 0 | 1 [7.7] | 0 |

CDAI, Crohn’s Disease Activity Index; EO, external opening; PDAI, Perianal Crohn’s Disease Activity Index; PFCD, perianal fistulizing Crohn’s disease; SES-CD, Simple Endoscopic Score for CD.
^a^Treatment failure was defined as the commencement or dose escalation of potentially effective non-study treatment for PFCD.

^b^CD non-responders were defined as participants who either had a baseline CDAI score of ≥220 and did not achieve a ≥70-point reduction in the CDAI score at any time up to and including week 10, or had a baseline CDAI score of <220 and an increase in the CDAI score of ≥100 from baseline, with a CDAI score of ≥220 at week 10.
^c^PFCD non-responders were defined as participants who met the following Perianal CD Activity Index [PDAI] symptom subscore criteria: ‘Discharge’ subscore of >1 and a ≥1-point increase from baseline at week 6 and week 10, or ‘Pain/restriction of activities’ subscore of >1 and a ≥1-point increase from baseline at week 6 and week 10. ^d^Worsening luminal disease was defined as a ≥100-point increase in CDAI score from the week 10 value, with a CDAI score of ≥220 at two consecutive visits.


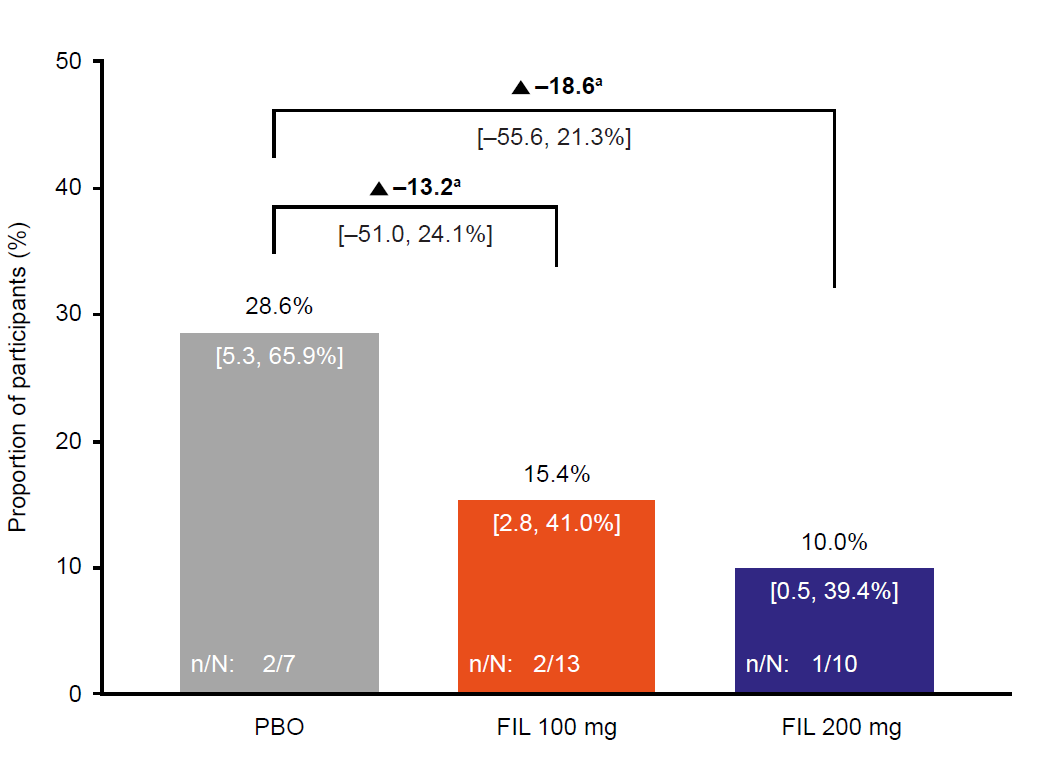


**Supplementary Figure 1.** Proctitis remission at week 24 [subset of the full analysis set].
Proportion of participants who achieved proctitis remission at week 24 [defined as a proctitis SES-CD score of 0 among participants who had moderately to severely active proctitis [proctitis SES-CD score of >2] at baseline]. Values in parentheses are 90% CI. ^a^Risk difference in proportions: non-responder imputation. CI, confidence interval; FIL, filgotinib; PBO, placebo; SES-CD, simple endoscopic score for Crohn’s disease.


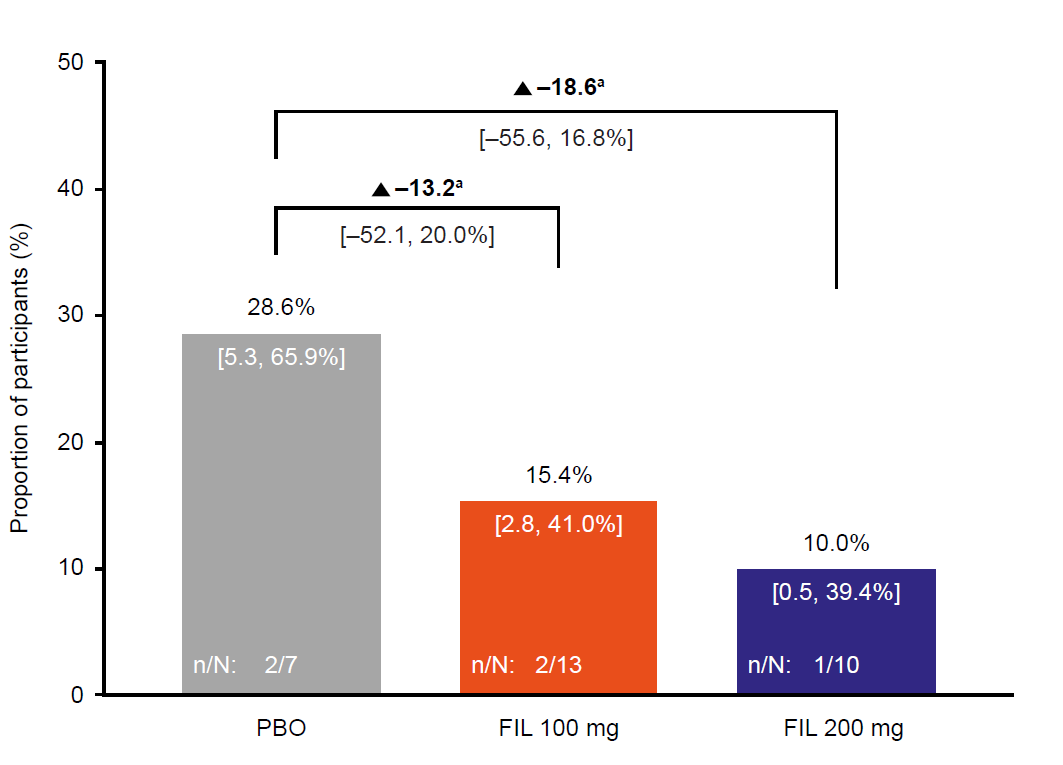


**Supplementary Figure 2.** Reduction of ≥50% in proctitis SES-CD score at week 24 [subset of the full analysis set].
Proportion of participants who achieved a ≥50% reduction in proctitis SES-CD score at week 24 among participants who had moderately to severely active proctitis [proctitis SES-CD score of >2] at baseline. Values in parentheses are 90% CI. ^a^Risk difference in proportions: non-responder imputation. CI, confidence interval; SES-CD, simple endoscopic score for Crohn’s disease.
